# The Role of Step Length and Step Frequency in the 400-meters Hurdles Performance

**DOI:** 10.5114/jhk/205439

**Published:** 2025-11-19

**Authors:** Nerea Casal-García, José Luis López-del Amo

**Affiliations:** 1 National Institute of Physical Education of Catalonia (INEFC), University of Lleida (UdL), La Seu d’Urgell, Spain.; 2 National Institute of Physical Education of Catalonia (INEFC), University of Barcelona (UB), Barcelona, Spain.

**Keywords:** performance analysis, athletics, step pattern, stride frequency

## Abstract

Running speed represents the optimal balance between stride frequency (SF) and stride length (SL), both crucial determinants of performance. In recent years, there has been a significant increase in performance in the 400-m hurdles (mH) for both men and women. The main purpose of this study was to assess whether SF and SL were significant indicators of performance in the 400 mH. Additionally, the study sought to determine whether the ratio of stride length to stride frequency (SL/SF) could serve as a more effective performance indicator compared to analysing SL and SF separately. A total of 511 individual performances from four major championships (the World Championships Doha 2019 and Oregon 2022, the Tokyo 2020 Olympics and the Munich 2022 European Championships) were analysed through video during competition. SL showed high to moderate correlations with performance in both sexes, especially in the second half of the race. However, SF did not correlate with final performance and showed only minor differences between groups. Elite athletes demonstrated a higher SL/SF ratio, as well as greater SL, with more pronounced differences in the second half of the race. Coaches should consider using the SL/SF ratio rather than SF alone to enhance its validity as a kinematic variable related to performance. The current top athletes exhibit higher values for SL and the SL/SF ratio, with particular emphasis during the second half of the event.

## Introduction

The 400-m hurdles (mH) is one of the most complex disciplines in athletics, demanding both exceptional speed endurance and precise hurdle technique (Przednowek et al., 2016; Quercetani, 2000). This event has a significant glycolytic component, leading to a progressive decrease in speed, which is characterised by a reduction in both step length (SL) and frequency (SF) (Cicchella, 2022; Gupta et al., 1999; Hanon et al., 2010; Zouhal et al., 2010).

Running speed is the product of SL and SF, requiring an optimal balance between them (Otsuka and Isaka, 2019; Salo et al., 2011). A step is defined as half of a running cycle, specifically, the phase from the contact of one foot to the contact of the opposite foot (Hunter et al., 2004). It should be emphasised that, although the product of SL and SF is equal to speed, and therefore increasing both would theoretically increase speed, they usually have an inversely proportional relationship during maximal efforts (Salo et al., 2011). The literature has sometimes been inconsistent on whether SF or SL is the more critical limiting factor for speed (Hunter et al., 2004; Mero et al., 1992). Recent findings suggest that the duty factor can also serve as a valuable indicator of running biomechanics, as it relates to external loading characteristics independently of step frequency, thereby offering additional insight beyond SF alone (Van der Meulen et al., 2024).

In the 400 mH, both the SF and SL cannot be understood without considering the rhythm. Placing 10 hurdles every 35 m requires athletes to adopt what is known as the hurdle rhythm, which is a key factor of performance in this event. Hurdle rhythm is determined by the number of steps taken between each hurdle (rhythmic unit), the speed of each interval based on the number of steps (step frequency), and the athlete's motor skills and physical conditioning (Iskra and Coh, 2011). In addition, laterality and coordination play a crucial role in the 400 mH. Apart from an athlete’s preference for their dominant leg, attacking hurdles with the left leg on the curve reduces the distance covered (Schiffer, 2012).

In the 400 m flat race, the highest SF is achieved between 50 m and 100 m, with a significant decrease occurring between the 250 m and the 400 m segment (Gajer et al., 2007; Hanon and Gajer, 2009). Moreover, it has been found that under fatigue conditions, the decrease is more pronounced in SF than in SL in 400-m sprinters (Mackala et al., 2024). This decline is also observed at a similar location in the 400 mH, where the first change in the rhythm typically occurs between the sixth and eighth rhythmic units among elite athletes (Guex, 2012; López and Casal, 2023). Velocity loss is caused by fatigue, due to the high anaerobic component in both events (Duffield et al., 2005; Hanon et al., 2010).

There is a lack of recent studies on SF and its derived variables in high-level 400 mH. Existing research primarily focusses on finals, often segregated by sex, and sometimes relies on television footage, which can lead to data loss due to changes in camera perspectives and the focus on top athletes, excluding those in the last positions. Furthermore, to our knowledge, only two studies have addressed this topic (Guex, 2012; Otsuka and Isaka, 2019). Although the relationship between SF and SL has been analysed in terms of 400 mH performance, no studies have directly compared these variables between sexes. This study provides highly relevant information on current 400 mH performance, including data from many of the top performances in recent years (such as the men’s world record and three previous women’s world records), representing the historical peak performance moment of the event. These data can be used by coaches to evaluate the significance of different variables in the athlete’s performance assessments. Therefore, the primary objective of this study was to determine whether SF and SL were key indicators of performance in the 400 mH. The secondary objective was to assess whether the ratio between SL and SF was a better performance indicator than these variables considered separately. We hypothesised that SL would be a crucial variable in performance; however, due to its characteristics, SF might not be as significant. Therefore, the SL/SF ratio might provide valuable insights into their relationship and its impact on performance in the 400 mH.

## Methods

### Participants

The sample included all participants in the 400 mH from four major championships: the 2019 World Championships in Doha, the 2020 Tokyo Olympics, the 2022 World Championships in Oregon, and the 2022 European Championships in Munich. A total of 511 individual performances (255 and 256 of women and men, respectively) were analysed. The average final performance for the female sample was 55.48 ± 1.42 s, and for the male sample, it was 49.47 ± 1.16 s (Table 1). The average age was 26.2 ± 3.8 years for women and 25.4 ± 3.4 years for men.

**Table 1:** Mean ± SD, minimum and maximum divided by groups and sex for the variables derived from step length and step frequency.

Variable	Men (n = 256)				Women (n = 255)			
	HP (n = 86)	IP (n = 86)	LP (n = 84)	Finalists (n = 32)	HP (n = 84)	IP (n = 85)	LP (n = 86)	Finalists (n = 31)
Performance (s)	48.26 ± 0.7 (45.94–49.06)	49.47 ± 0.21 (49.08–9.83)	50.69 ± 0.77 (49.86–53.07)	48.01 ± 1.06 (45.94–50.46)	54.00 ± 0.88 (50.68–54.91)	55.42 ± 0.30 (54.93–55.93)	56.98 ± 0.83 (55.94–60.36)	53.84 ± 1.41 (50.68–56.02)
SL 400 m (m)	2.38 ± 0.06 (2.12–2.52)	2.31 ± 0.08 (2.11–2.48)	2.26 ± 0.09 (2.06–2.40)	2.38 ± 0.07 (2.20–2.51)	2.12 ± 0.05 (2.00–2.23)	2.07 ± 0.06 (1.93–2.22)	2.02 ± 0.05 (1.89–2.12)	2.12 ± 0.06 (1.96–2.23)
SL in the first 200 m (m)	2.46 ± 0.10 (2.13–2.63)	2.40 ± 0.12 (2.13–2.63)	2.36 ± 0.13 (2.11–2.60)	2.45 ± 0.08 (2.26–2.63)	2.16 ± 0.06 (2.04–2.30)	2.12 ± 0.07 (1.98–2.29)	2.08 ± 0.05 1.95–2.15)	2.16 ± 0.07 (1.98–2.30)
SL in the second 200 m (m)	2.31± 0.11 (2.12–2.52)	2.23 ± 0.10 (2.00–2.50)	2.16 ± 0.10 (1.80–2.35)	2.31 ± 0.10 (2.11–2.52)	2.09 ± 0.06 (1.94–2.22)	2.01 ± 0.07 (1.87–2.16)	1.96 ± 0.06 (1.79–2.11)	2.09 ± 0.07 (1.91–2.22)
Difference between SL first and second 200 m (m)	0.14 ± 0.16 (−0.09–0.38)	0.18 ± 0.15 (−0.09–0.43)	0.20 ± 0.14 (−0.16–0.61)	0.14 ± 0.13 (−0.02–0.43)	0.07 ± 0.05 (−0.06–0.18)	0.11 ± 0.05 (0.00–0.21)	0.12 ± 0.05 (0.02–0.25)	0.07 ± 0.06 (−0.06–0.19)
SF 400 m (Hz)	3.48 ± 0.08 (3.32–3.85)	3.50 ± 0.12 (3.26–3.84)	3.50 ± 0.13 (3.22–3.87)	3.50 ± 0.06 (3.36–3.65)	3.37 ± 0.41 (3.29–3.67)	3.48 ± 0.19 (3.27–3.71)	3.49 ± 0.09 (3.30–3.71)	3.50 ± 0.09 (3.33–3.68)
SF in the first 200 m (Hz)	3.60 ± 0.14 (3.21–4.03)	3.58 ± 0.17 (3.14–4.03)	3.59 ± 0.18 (3.19–4.00)	3.65 ± 0.12 (3.40–3.89)	3.65 ± 0.09 (3.38–3.82)	3.65 ± 0.12 (3.39–3.93)	3.66 ± 0.10 (3.47–3.96)	3.67 ± 0.11 (3.42–3.93)
SF in the second 200 m (Hz)	3.38 ± 0.14 (3.13–3.88)	3.44 ± 0.16 (3.05–3.84)	3.43 ± 0.16 (3.02–4.08)	3.38 ± 0.09 (3.21–3.52)	3.35 ± 0.09 (3.17–3.57)	3.37 ± 0.12 (3.13–3.61)	3.34 ± 0.11 (3.10–3.59)	3.35 ± 0.10 (3.13–3.52)
Difference in SF between the first and the second 200 m (Hz)	−0.22 ± 0.23 (−0.54–0.64)	−0.14 ± 0.22 (−0.52–0.62)	−0.16 ± 0.22 (−0.52–0.48)	−0.27 ± 0.17 (−0.51–0.08)	−0.31 ± 0.09 (-0.55– −0.11)	−0.28 ± 0.11 (−0.52–0.10)	−0.32 ± 0.10 (−0.68– −0.14)	−0.32 ± 0.09 (-0.52– −0.17)
SL/SF ratio	0.68 ± 0.03 (0.55–0.76)	0.66 ± 0.04 (0.55–0.76)	0.66 ± 0.05 (0.53–0.73)	0.68 ± 0.03 (0.61–0.74)	0.65 ± 0.12 (0.54–0.68)	0.60 ± 0.06 (0.52–0.68)	0.58 ± 0.03 (0.51–0.64)	0.61 ± 0.03 (0.53–0.67)
SL/SF first 200 m ratio	0.68 ± 0.05 (0.53–0.87)	0.68 ± 0.07 (0.53–0.88)	0.66 ± 0.07 (0.53–0.80)	0.67 ± 0.04 (0.59–0.77)	0.59 ± 0.03 (0.54–0.68)	0.58 ± 0.04 (0.50–0.67)	0.57 ± 0.03 (0.51–0.62)	0.59 ± 0.03 (0.50–0.67)
SL/SF second 200 m ratio	0.69 ± 0.06 (0.51–0.80)	0.65 ± 0.06 (0.52–0.81)	0.63 ± 0.05 (0.44–0.77)	0.69 ± 0.04 (0.60–0.78)	0.63 ± 0.03 (0.55–0.69)	0.60 ± 0.04 (0.52–0.69)	0.59 ± 0.03 (0.50–0.67)	0.62 ± 0.03 0.55–0.68
% of hurdles attacked with the left leg (%)	52.33 ± 39.25 (0.00–100.00)	63.72 ± 29.86 (0.00–100.00)	66.07 ± 27.64 (0.00–100.00)	52.50 ± 39.84 (0.00–100.00)	49.88 ± 33.67 (10.00–100.00)	53.76 ± 30.39 (00.00–100.00)	57.33 ± 26.72 (10.00–100.00)	49.35 ± 32.76 (10.00–100.00)
% of curved hurdles attacked with the left leg (%)	52.79 ± 40.57 (0.00–100.00)	63.95 ± 29.08 (0.00–100.00)	62.62 ± 26.85 (0.00–100.00)	52.50 ± 41.81 (0.00–100.00)	49.29 ± 36.79 (0.00–100.00)	52.47 ± 29.76 (00.00–100.00)	57.44 ± 27.58 (0.00–100.00)	49.03 ± 34.95 (0.00–100.00)

SL: Step length, SF: Step frequency, HP: high performance group, IP: intermediate performance group, LP: low performance group

Performances were categorized into three groups based on final times. Final results were divided equally by performance levels. In the event of identical times across groups, both were placed in the same group to avoid duplication. The high-performance (HP) group included 84 women and 86 men, the intermediate-performance group (IP) included 85 women and 86 men, and the low-performance (LP) group included 84 men and 85 women. To further analyse finalists, a secondary analysis was performed, categorising participants into finalists (Group A), comprising 32 men and 31 women, and semi-final and preliminary round participants (Group B), comprising 224 men and 224 women. Of the finalists, 29 (90.6%) men and 24 (77.4%) women belonged to the HP group, 1 (3.1%) man and 6 (19.4%) women to the IP group and 2 (6.3%) men and 1 (3.2%) woman to the LP group.

### Design and Procedures

**Figure 1:**
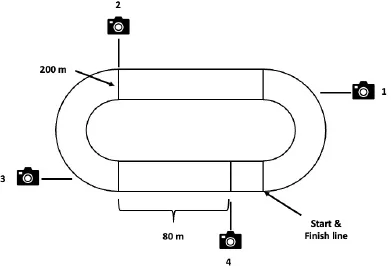
Location of the cameras in the stadium: Camera 1 at the end of the first curve; Camera 2 at the 200-m mark of lane 5; Camera 3 at the end of the second curve; camera 4 20 m before the lane 1 start line.

Since 2D video analysis is a commonly used tool for calculating kinematic variables in sports (Mundt et al., 2024), the data were collected using four HD Sony R6 cameras (60 Hz) placed in the stadium stands (Figure 1): one camera at the end of the first curve, one at the 200-m mark on lane 5, one at the end of the second curve, and one at the 80-m mark on the home straight. The placement of cameras remained as consistent as possible across the championships, considering architectural differences among stadiums. However, the following criteria were maintained for each camera: all cameras must have captured all athletes throughout every race. Cameras positioned at the end of the first and second curves must have had a clear view of the back straight and the main straight, respectively, ensuring that the athletes did not block each other from the camera's perspective. This setup guaranteed that all athlete’s steps and lead legs were recorded by the cameras. The study was observational and did not interfere with athletes’ performance, thus informed consent was not required. The final result was obtained from the official results, measured by SEIKO (Tokyo, Japan) and OMEGA (Biel, Switzerland).

The video files were imported into Kinovea (0.9.5), a reliable tool for analysing spatial and temporal variables (Nor Adnan et al., 2018), and analysed by three independent experts. Race time for the first half was obtained from camera 2, using the starting gun as a reference. Steps were counted from multiple cameras and totalled for each race half. For the first 200 m, steps were counted directly from camera 2, eliminating the need for indirect calculations. The number of steps was counted for each interval between hurdles (H) (from the start to H1, H1–H2, H2–H3, H3–H4, H4–H5, H5–H6, H6–H7, H7–H8, H8–H9, H9–H10, and H10 to the finish line). Step length was calculated by dividing the total distance by the number of steps, with an average computed for each interval. Step frequency was determined by dividing the number of steps per interval by the interval time. The collected data were processed using Microsoft Excel (16.86, Microsoft). The study was approved by the Ethics Committee of the Catalan Sports Council, Barcelona, Spain (approval code: 026/CEICGC/2021; approval date: 23 July 2021).

### Statistical Analysis

Descriptive statistics for each variable, including mean, standard deviation, minimum, and maximum, are presented in Table 1. Normality was assessed using the Kolmogorov-Smirnov test. A one-way analysis of variance (ANOVA) was used to determine differences in SL, SF, and the SL/SF ratio among the HP, IP, and LP groups. Additionally, a Student's *t*-test was conducted to compare these variables between finalists (Group A) and other participants (Group B). Post-hoc comparisons were conducted using Bonferroni correction when applicable. Homogeneity of variances was tested with the Levene’s test, and Welch's correction was applied when the assumption was violated. The effect sizes were calculated using η^2^, and interpreted as follows (Fritz et al., 2012): trivial (< 0.01), small (≥ 0.01), moderate (≥ 0.06), and large (≥ 0.14). Cohen’s *d* thresholds were set and interpreted as (Fritz et al., 2012): trivial (0–0.2), small (0.2–0.8), moderate (0.8–1.2), large (1.2–2), and very large (>2). Statistical analysis was performed using JASP (0.18.3, University of Amsterdam).

## Results

The descriptive statistics of the analysed variables are shown in Tables 1 and 2. A similar trend was observed for both men and women. High-performance athletes exhibited higher average SL because they took fewer steps. These differences were more pronounced in the second half of the race. SF was higher among lower-performing athletes for both sexes. However, the SL/SF ratio was greater in high-performing athletes. The finalists achieved similar values in all variables compared to the HP group. Regarding the use of the left leg for hurdle clearance, both men and women showed a lower number of hurdles cleared with this leg among finalists and higher-level athletes.

**Table 2:** Mean ± SD, minimum and maximum of each interval for the male sample for the variables SL, SF and the SL/SF ratio.

	SL				SF				Ratio SL/SF			
	HP (n = 86)	IP (n = 86)	LP (n = 84)	Finalists (n = 32)	HP (n = 86)	IP (n = 86)	LP (n = 84)	Finalists (n = 32)	HP (n = 86)	IP (n = 86)	LP (n = 84)	Finalists (n = 32)
H1–H2	2.69 ± 0.04 (2.33–2.69)	2.63 ± 0.10 (2.33–2.69)	2.58 ± 0.14 (2.33–2.69)	2.69 ± 0.03 (2.50–2.69)	3.56 ± 0.10 (3.31–3.92)	3.53 ± 0.13 (3.31–3.93)	3.57 ± 0.18 (3.27–4.02)	3.60 ± 0.09 (3.39–3.76)	0.76 ± 0.03 (0.60–0.81)	0.75 ± 0.05 (0.59–0.81)	0.73 ± 0.07 (0.58–0.82)	0.75 ± 0.02 (0.68–0.79)
H2–H3	2.69 ± 0.06 (2.33–2.92)	2.63 ± 0.11 (2.33–2.92)	2.58 ± 0.14 (2.33–2.69)	2.69 ± 0.05 (2.50–2.92)	3.48 ± 0.09 (3.26–3.87)	3.47 ± 0.14 (3.18–3.88)	3.48 ± 0.17 (3.17–3.88)	3.51 ± 0.10 (3.33–3.70)	0.77 ± 0.03 (0.60–0.89)	0.76 ± 0.06 (0.60–0.92)	0.74 ± 0.07 (0.60–0.85)	0.77 ± 0.03 (0.70–0.88)
H3–H4	2.71 ± 0.08 (2.33–2.92)	2.63 ± 0.11 (2.33–2.92)	2.58 ± 0.14 (2.33–2.69)	2.72 ± 0.09 (2.50–2.92)	3.40 ± 0.11 (3.08–3.85)	3.38 ± 0.14 (3.08–3.83)	3.40 ± 0.18 (3.07–3.95)	3.43 ± 0.12 (3.13–3.64)	0.80 ± 0.05 (0.61–0.95)	0.78 ± 0.06 (0.61–0.95)	0.76 ± 0.08 (0.59–0.88)	0.80 ± 0.05 (0.69–0.93)
H4–H5	2.71 ± 0.08 (2.33–2.92)	2.63 ± 0.11 (2.33–2.92)	2.57 ± 0.14 (2.33–2.69)	2.72 ± 0.09 (2.50–2.92)	3.30 ± 0.12 (2.97–3.77)	3.29 ± 0.13 (2.98–3.74)	3.30 ± 0.19 (2.97–3.80)	3.33 ± 0.11 (3.05–3.58)	0.82 ± 0.05 (0.62–0.98)	0.80 ± 0.06 (0.62–0.98)	0.78 ± 0.08 (0.61–0.91)	0.82 ± 0.05 (0.72–0.96)
H5–H6	2.68 ± 0.08 (2.33–2.92)	2.58 ± 0.14 (2.33–2.92)	2.50 ± 0.13 (2.33–2.69)	2.67 ± 0.08 (2.50–2.92)	3.21 ± 0.10 (2.95–3.65)	3.25 ± 0.17 (2.91–3.63)	3.27 ± 0.16 (2.97–3.61)	3.25 ± 0.09 (3.03–3.58)	0.84 ± 0.04 (0.64–0.99)	0.80 ± 0.08 (0.64–1.00)	0.77 ± 0.07 (0.65–0.91)	0.82 ± 0.04 (0.72–0.94)
H6–H7	2.62 ± 0.11 (2.33–2.69)	2.50 ± 0.11 (2.33–2.69)	2.44 ± 0.10 (2.19–2.69)	2.62 ± 0.10 (2.33–2.69)	3.16 ± 0.12 (2.93–3.53)	3.21 ± 0.14 (2.87–3.50)	3.23 ± 0.14 (2.86–3.58)	3.25 ± 0.09 (3.10–3.47)	0.83 ± 0.06 (0.66–0.92)	0.78 ± 0.07 (0.67–0.94)	0.76 ± 0.06 (0.61–0.94)	0.83 ± 0.05 (0.69–0.89)
H7–H8	2.56 ± 0.12 (2.33–2.69)	2.46 ± 0.12 (2.33–2.69)	2.38 ± 0.10 (2.19–2.50)	2.56 ± 0.14 (2.33–2.69)	3.12 ± 0.11 (2.91–3.42)	3.18 ± 0.16 (2.81–3.43)	3.19 ± 0.15 (2.78–3.57)	3.16 ± 0.09 (3.03–3.44)	0.82 ± 0.07 (0.68–0.92)	0.78 ± 0.08 (0.68–0.96)	0.75 ± 0.06 (0.61–0.90)	0.82 ± 0.07 (0.70–0.92)
H8–H9	2.50 ± 0.13 (2.19–2.69)	2.40 ± 0.10 (2.19–2.69)	2.34 ± 0.10 (2.06–2.50)	2.51 ± 0.15 (2.19–2.69)	3.11 ± 0.13 (2.91–3.52)	3.15 ± 0.14 (2.78–3.59)	3.14 ± 0.14 (2.78–3.59)	3.14 ± 0.11 (2.91–3.39)	0.81 ± 0.07 (0.62–0.93)	0.76 ± 0.07 (0.63–0.95)	0.75 ± 0.06 (0.57–0.90)	0.81 ± 0.07 (0.67–0.93)
H9–H10	2.47 ± 0.14 (2.19–2.69)	2.38 ± 0.09 (2.19–2.69)	2.32 ± 0.10 (2.06–2.50)	2.45 ± 0.14 (2.19–2.69)	3.07 ± 0.14 (2.75–3.41)	3.11 ± 0.13 (2.82–3.52)	3.09 ± 0.13 (2.82–3.52)	3.09 ± 0.13 (2.84–3.41)	0.81 ± 0.08 (0.65–0.98)	0.77 ± 0.66 (0.64–0.97)	0.75 ± 0.06 (0.58–0.89)	0.79 ± 0.08 (0.68–0.95)

SL: Step length, SF: Step frequency, HP: high performance group, IP: intermediate performance group, LP: low performance group

Regarding the differences between groups for SL, significant differences were found in almost all groups in SL for both men (F(2,254) = 62.914, *p* < 0.001,η^2^ = 0.310, large) and women (F(2,252) = 82.487, *p* < 0.001, η^2^ = 0.396, large). For SL over 400 mH, the effect size was large between the HP and LP groups (*p* < 0.001, *d* = 1.61), and moderate between the HP and the IP group (*p* < 0.001, *d* = 0.91) and between the IP and LP groups (*p* < 0.001, *d* = 0.70). For SL in the first 200 m, the differences were moderate between the HP and LP groups (*p* < 0.001, *d* = 0.80) and small between the HP and IP groups (*p* = 0.01, *d* = 0.45), with no significant differences between the IP and LP groups (*p* = 0.15). In the second 200 m, the differences were large between the HP and LP groups (*p* < 0.001, *d* = 1.46) and moderate between the HP and IP groups and between the IP and LP groups (*d* = 0.80 and *d* = 0.66, respectively). No significant differences were found between the 200-m segments (*p* > 0.05). In the female sample, significant differences were observed between all groups for the three variables (*p* < 0.001). For SL over 400 mH, the effect size was moderate between the IP group and the HP and LP groups (*p* < 0.001, *d* = 1.03 and *p* < 0.001, *d* = 0.93, respectively) and large between the HP and LP groups (*p* < 0.001, *d* = 1.96). For SL in the first 200-m interval, the differences remained moderate between the IP group and the HP and LP groups (*p* < 0.001, *d* = 0.63 and *p* < 0.001, *d* = 0.76, respectively) and large between the HP and LP groups (*p* < 0.001, *d* = 1.39). For SL in the second 200-m interval, the differences between the groups were more pronounced, with moderate differences between the IP and LP groups (*p* < 0.001, *d* = 0.90), large differences between the HP and IP groups (*p* < 0.001, *d* = 1.24), and very large differences between the HP and LP groups (*p* < 0.001, *d* = 2.13). In contrast to the male sample, there were significant differences in the change in SL between the first and second 200-m segments between female athletes. Specifically, significant differences were observed between the HP group and the IP group (*p* < 0.001, *d* = −0.81, moderate) as well as between the HP group and the LP group (*p* < 0.001, *d* = −1.07, large).

When analysing each interval independently, significant differences in SL between groups were also found. In the male sample, significant differences were found for the following intervals: H1–H2 (*p* < 0.001, *d* = 1.08, moderate) between LP and HP groups; H2–H3 between HP and IP groups (*p* < 0.001, *d* = 0.59, small) and between HP and LP groups (*p* < 0.001, *d* = 1.08, large); H3–H4 among HP, IP and LP groups (*p* < 0.001, *d* = 0.68, small; *p* < 0.001, *d* = 1.18, large); H4–H5 among HP, IP and LP groups (*p* < 0.001, *d* = 0.73, small; *p* < 0.001, *d* = 1.23, large ); H5–H6 among HP, IP and LP groups (*p* < 0.001, *d* = 0.90, moderate; *p* < 0.001, *d* =1.55, large, respectively) and between IP and LP groups (*p* < 0.001, *d* = 0.65, small); H6–H7 among HP, IP and LP groups (*p* < 0.001, *d* = 1.04, moderate; *p* < 0.001, *d* = 1.63, large, respectively) and between IP and LP groups (*p* < 0.001, *d* = 0.58, small); H7–H8 among HP, IP and LP groups (*p* < 0.001, *d* = 0.91, moderate; *p* < 0.001, *d* = 1.60, large, respectively) and between IP and LP groups (*p* < 0.001, *d* = 0.69, small); H8–H9 among HP, IP and LP groups (*p* < 0.001, *d* = 0.87, moderate; *p* < 0.001, *d* = 1.39, large, respectively); and at the H9–H10 interval among HP, IP and LP groups (*p* < 0.001, *d* = 0.77, small; *p* < 0.001, *d* = 1.32, large, respectively). Significant differences were also found in the female sample in the following intervals: H1–H2 among HP, IP and LP groups (*p* < 0.001, *d* = 0.60, small; *p* < 0.001, *d* = 1.06, moderate, respectively); H2–H3 among HP, IP and LP groups (*p* < 0.001, *d* = 0.66, small; *p* < 0.001, *d* = 1.14, moderate, respectively); H3–H4 among HP, IP and LP groups (*p* < 0.05, *d* = 0.50, small *p* < 0.001, *d* = 0.99, moderate, respectively); H4–H5 among LP, HP and IP groups (*p* < 0.001, *d* = 1.20, large; *p* < 0.001, *d* = 0.74, moderate, respectively); H5–H6 among HP, IP and LP groups (*p* < 0.001, *d* = 0.87, moderate; *p* < 0.001, *d* = 1.42, large, respectively); H6–H7 among HP, IP and LP groups (*p* < 0.001, *d* = 0.87, moderate; *p* < 0.001, *d* = 1.65, large, respectively) and between IP and LP group (*p* < 0.001, *d* = 0.78, small); H7–H8 among HP, IP and LP groups (*p* < 0.001, *d* = 1.04, moderate; *p* < 0.001, *d* = 1.74, large, respectively) and between IP and LP groups (*p* < 0.001, *d* = 0.69, small); H8–H9 among HP, IP and LP groups (*p* < 0.001, *d* = 1.01, moderate; *p* < 0.001, *d* = 1.85, large, respectively) and between IP and LP groups (*p* < 0.001, *d* = 0.85, moderate); and in the H9–H10 interval among HP, IP and LP groups (*p* < 0.001, *d* = 1.21, large; *p* < 0.001, *d* = 2.15, very large, respectively) and between IP and LP groups (*p* < 0.001, *d* = 0.94, large).

In contrast, differences in SF were not significant for any of the segments analysed among the groups (*p* > 0.05). For the male sample, the ANOVA results were as follows: SF for the 400 mH race (F_(2,254)_ = 2.025, *p* = 0.135, η^2^ = 0.016); SF for the first 200-m segment (F_(2,254)_ = 0.537, *p* = 0.585, η^2^ = 0.004), and SF for the second 200-m segment (F_(2,254)_ = 3.280, *p* = 0.039, η^2^ = 0.025), with Bonferroni post-hoc comparisons revealing no significant differences among groups (*p* > 0.05). No differences were found between SF in the first and the second 200-m interval. For the female sample, the ANOVA results were as follows: SF for 400 mH race (F_(2,252)_ = 0.173, *p* = 0.841, η^2^ = 0.002), SF for the first 200 m (F_(2,252)_ = 0.322, *p* = 0.725, η^2^ = 0.003), SF for the second 200 m (F_(2,252)_ = 3.34, *p* = 0.260, η^2^ = 0.011) and the SF difference between the first and the second 200-m segment (F_(2,252)_ = 3.31, *p* = 0.04, η^2^ = 0.003), with a small difference between IP and LP groups (*p* = 0.04, *d* = 0.39).

When analysing each interval independently, small significant differences were found only in some intervals in the male sample: H7–H8 among HP, IP and LP groups (*p* = 0.02, *d* = −0.42; *p* < 0.1, *d* = −0.53, respectively), and at the H6–H7 interval among HP, IP and LP groups (*p* = 0.02, *d* = −0.42; *p* < 0.01, *d* = −0.55, respectively). In contrast, no such differences were found in the female sample.

Regarding the SL/SF ratio, in the male sample, significant differences were found for the 400 mH between the high-performance (HP) and intermediate-performance (IP) groups (*p* = 0.001, *d* = 0.53), with a small effect, and between the HP and low-performance (LP) groups (*p* < 0.001, *d* = 0.89), with a moderate effect. No differences were observed among the groups for the SL/SF ratio in the first 200 m. In the second 200 m, significant differences were found between the HP group and the IP and LP groups (*p* < 0.001, *d* = 0.63 and *p* <0.001, *d* = 0.96), with moderate effects. In the female sample, similar differences were observed. For the 400 mH, significant differences with small and moderate effects were found between the HP group and the IP and LP groups (*p* < 0.001, *d* = 0.59 and *p* < 0.001, *d* = 0.82). In the first 200-m segment, significant differences were observed between the LP group and the IP and HP groups (*p* = 0.01, *d* = 0.44 and *p* < 0.001, *d* = 0.74), with small and moderate effects. Finally, larger differences were found in the SL/SF ratio for the second 200 m, with moderate effects between the HP group and the IP and LP groups (*p* < 0.001, *d* = 0.74 and *p* < 0.001, *d* = 1.11, respectively).

When the intervals were analysed independently, significant differences were found between the HP and LP groups in all intervals, and between the HP and IP groups in the second half of the race. In the male sample, significant differences were found for the following intervals and groups: H1–H2 between HP and LP groups (*p* < 0.001, *d* = 0.57, small); H2–H3 between HP and LP groups (*p* < 0.01, *d* = 0.53, small); H3–H4 between HP and LP groups (*p* < 0.001, *d* = 0.58, small); H4–H5 between HP and LP groups (*p* < 0.001, *d* = 0.62, small); H5–H6 among HP, IP and LP groups (*p* < 0.001, *d* = 0.57, small; *p* < 0.001, *d* = 0.97, moderate, respectively); H6–H7 among HP, IP and LP groups (*p* < 0.001, *d* = 0.76, small; *p* < 0.001, *d* = 1.13, moderate, respectively); H7–H8 among HP, IP and LP groups (*p* < 0.001, *d* = 0.67, small; *p* < 0.001, *d* = 1.08, moderate, respectively) and a small difference between IP and LP groups (*p* = 0.02, *d* = 0.42); H8–H9 among HP, IP and LP groups (*p* < 0.001, *d* = 0.63, small; *p* < 0.001, *d* = 0.88, moderate, respectively); and at the H9–H10 interval among HP, IP and LP groups (*p* < 0.001, *d* = 0.58, small; *p* < 0.001, *d* = 0.79, small, respectively). However, in the female sample, no significant differences were found in the H1–H2 and H3–H4 intervals, with differences observed in the following intervals: H2–H3 between HP and LP groups (*p* < 0.001, *d* = 0.59, small); H4–H5 between HP and LP groups (*p* < 0.001, *d* = 0.66, small); H5–H6 between HP and LP groups (*p* < 0.001, *d* = 0.83, moderate); H6–H7 between HP and LP groups (*p* < 0.001, *d* = 1.00, moderate); H7–H8 among HP, IP and LP groups (*p* < 0.001, *d* = 0.62, small; *p* < 0.001, *d* = 1.02, moderate, respectively) and a small difference between IP and LP group (*p* = 0.03, *d* = 0.40); H8-H9 between HP and IP and LP groups (*p* < 0.001, *d* = 0.60, small; *p* < 0.001, *d* = 1.02, moderate, respectively) and a small difference between IP and LP groups (*p* = 0.03, *d* = 0.42); and at the H9–H10 interval among HP, IP and LP groups (*p* < 0.001, *d* = 0.78, small; *p* < 0.001, *d* = 1.19, moderate, respectively) and a small difference between IP and LP groups (*p* = 0.02, *d* = 0.41).

**Figure 2:**
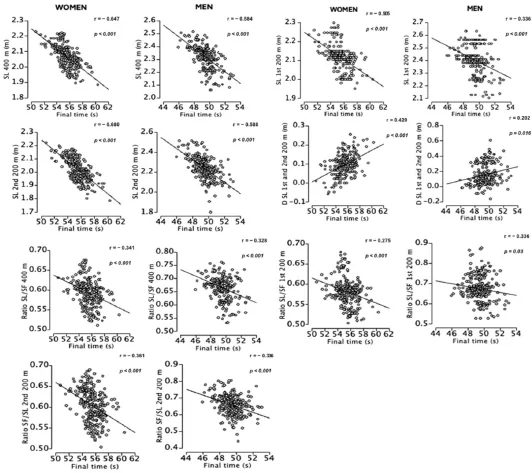
Pearson correlation of statistically significant variables, separated by sex (women: n = 255 and men: n = 256). SL = Step Length; SF = Step Frequency; D = Difference. Variables with p < 0.05 are considered significantly correlated

Regarding the correlations with the final performance (Figure 2), in the male sample, SL during the race showed an inverse proportional correlation with the final performance (r = −0.58, *p* < 0.001, moderate), as did SL in the first 200-m interval (r = −0.34, *p* < 0.001, weak) and in the second 200-m interval (r = −0.59, *p* < 0.001, moderate). The difference in SL between the two 200-m segments was also significant (r = 0.20, *p* = 0.016, weak). The SL/SF ratio showed a statistically significant inverse correlation for the entire race (r = −0.32, *p* < 0.001, weak) and in the second 200-m interval (r = −0.38, *p* < 0.001, weak), but not for the first 200 m (r = −0.12, *p* = 0.05). In the female sample, SL exhibited a statistically significant inverse correlation with final performance throughout the race (r = −0.65, *p* < 0.001, strong), in the first 200-m interval (r = −0.51, *p* < 0.001, moderate), and in the second 200-m interval (r = −0.68, *p* < 0.001, strong). A significant correlation in the difference between the two 200-m segments with respect to SL was also found (r = 0.42, *p* < 0.001, moderate). The SL/SF ratio showed a statistically significant inverse correlation throughout the race (r = −0.34, *p* < 0.001, weak), in the first 200 m (r = −0.27, *p* < 0.001, weak), and in the second 200-m interval (r = −0.36, *p* < 0.001, weak). SF did not show significant correlations or showed trivial correlations: average SF (r = −0.01, *p* = 0.87; r = −0.12, *p* = 0.05), average SF in the first 200 m (r = −0.06, *p* = 0.30; r = −0.02, *p* = 0.74), average SF in the second 200 m (r = 0.06, *p* = 0.35; r = −0.15, *p* = 0.02); and the SF difference between the first and the second 200-m interval (r = 0.09, *p* = 0.16; r = 0.14, *p* = 0.02) in men and women, respectively. No correlations were found between the number of hurdles attacked with the left leg during the race or in the curve.

**Figure 3:**
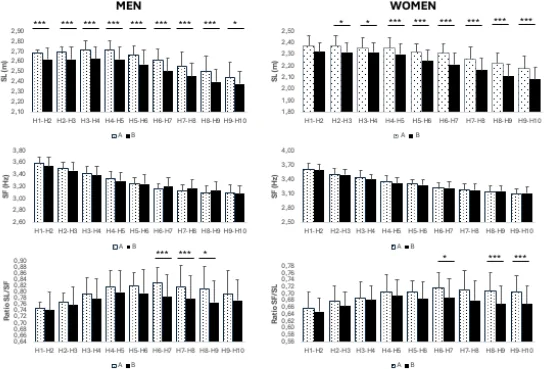
. Results of the Student’s *t*-test for SL, SF and the SF/SL ratio by interval between finalists (A) and athletes participating in heats and semifinals (B) divided by sex. SF: step frequency, SL: step length; * p < 0.05, ** p < 0.01, *** p < 0.001

The descriptive statistics of SL, SF, and the SL/SF ratio for each hurdle interval are presented in Tables 2 and 3 for men and women, respectively. The differences between groups A and B are shown in Figure 3. In the male sample, differences in SL were found in all intervals between groups A and B. Moderate differences were found in the following intervals: H3–H4 (*p* < 0.001, *d* = 0.85), H4–H5 (*p* < 0.001, *d*=0.88), H5–H6 (*p* < 0.01, *d* = 0.83), H6–H7 (*p* < 0.01, *d* = 0.99), and H8–H9 (*p* < 0.001, *d* = 0.80). The differences between the other intervals were small (*d* < 0.80). Regarding SF, no significant differences were found. In contrast with SL, only small differences were found for the SL/SF ratio in the following intervals: H6–H7 (*p* < 0.001, *d* = 0.72), H8–H9 (*p* < 0.5, *d* = 0.54) and H8–H9 (*p* < 0.5, *d* = 0.61). However, in the female sample, no significant differences were found in the H1–H4 intervals (*p* > 0.05) regarding the SL. Moderate differences were found in H4–H5 (*p* < 0.001, *d* = 0.97), H6–H7 (*p* < 0.001, *d* = 1.04), H7–H8 (*p* < 0.001, *d* = 1.13) and H9–H10 (*p* < 0.01, *d* = 0.97) intervals. Similarly to the male sample, no significant differences were found in SF between groups A and B. In contrast to SL, only small differences were found for the SL/SF ratio, as in the male sample, in H6–H7 (*p* < 0.5, *d* = 0.59), H8–H9 (*p* < 0.001, *d* = 0.72) and H9–H10 (*p* < 0.001, *d* = 0.68) intervals.

**Table 3:** Mean ± SD, minimum and maximum of each interval for the female sample for the variables SL, SF and the SL/SF ratio.

	SL				SF				Ratio SL/SF			
	HP (n = 86)	IP (n = 86)	LP (n = 85)	Finalists (n = 32)	HP (n = 86)	IP (n = 86)	LP (n = 85)	Finalists (n = 32)	HP (n = 86)	IP (n = 86)	LP (n = 85)	Finalists (n = 32)
H1–H2	2.37 ± 0.07 (2.19–2.50)	2.33 ± 0.08 (2.19–2.50)	2.29 ± 0.07 (2.19–2.50)	2.37 ± 0.09 (2.19–2.50)	3.60 ± 0.12 (3.31–3.84)	3.59 ± 0.13 (3.28–3.87)	3.58 ± 0.11 (3.22–3.72)	3.61 ± 0.13 (3.36–3.84)	0.66 ± 0.04 (0.57–0.76)	0.65 ± 0.04 (0.57–0.76)	0.64 ± 0.04 (0.57–0.76)	0.66 ± 0.05 (0.57–0.74)
H2–H3	2.37 ± 0.07 (2.19–2.50)	2.32 ± 0.08 (2.19–2.50)	2.29 ± 0.07 (2.19–2.33)	2.37 ± 0.09 (2.19–2.50)	3.50 ± 0.10 (3.22–3.72)	3.50 ± 0.11 (3.20–3.74)	3.49 ± 0.11 (3.28–3.81)	3.51 ± 0.12 (3.29–3.72)	0.68 ± 0.04 (0.60–0.78)	0.66 ± 0.04 (0.59–0.78)	0.66 ± 0.04 (0.57–0.71)	0.68 ± 0.05 (0.59–0.76)
H3–H4	2.36 ± 0.08 (2.19–2.50)	2.32 ± 0.08 (2.19–2.50)	2.29 ± 0.07 (2.06–2.33)	2.36 ± 0.08 (2.19–2.50)	3.43 ± 0.11 (3.14–3.70)	3.41 ± 0.12 (3.13–3.72)	3.40 ± 0.11 (3.19–3.75)	3.45 ± 0.13 (3.13–3.72)	0.69 ± 0.04 (0.60–0.80)	0.68 ± 0.05 (0.59–0.80)	0.67 ± 0.04 (0.57–0.73)	0.69 ± 0.05 (0.59–0.80)
H4–H5	2.36 ± 0.09 (2.06–2.50)	2.32 ± 0.08 (2.19–2.50)	2.25 ± 0.08 (2.06–2.33)	2.36 ± 0.08 (2.19–2.50)	3.33 ± 0.12 (3.05–3.66)	3.32 ± 0.11 (3.07–3.60)	3.33 ± 0.11 (3.13–3.72)	3.36 ± 0.12 (3.09–3.60)	0.71 ± 0.05 (0.56–0.82)	0.70 ± 0.05 (0.61–0.82)	0.68 ± 0.05 (0.55–0.75)	0.71 ± 0.05 (0.61–0.81)
H5–H6	2.32 ± 0.06 (2.06–2.50)	2.25 ± 0.09 (2.06–2.33)	2.20 ± 0.09 (2.06–2.33)	2.33 ± 0.07 (2.19–2.50)	3.27 ± 0.09 (3.09–3.52)	3.29 ± 0.13 (3.08–3.62)	3.29 ± 0.13 (3.05–3.56)	3.30 ± 0.09 (3.16–3.46)	0.71 ± 0.03 (0.59–0.78)	0.68 ± 0.05 (0.57–0.76)	0.67 ± 0.05 (0.58–0.77)	0.70 ± 0.03 (0.63–0.78)
H6–H7	2.30 ± 0.08 (2.06–2.50)	2.26 ± 0.09 (2.06–2.33	2.10 ± 0.08 (1.94–2.33)	2.31 ± 0.09 (2.06–2.50)	3.21 ± 0.10 (3.04–3.56)	2.23 ± 0.14 (2.96–3.51)	3.24 ± 0.12 (3.00–3.52)	3.23 ± 0.10 (3.06–3.47)	0.72 ± 0.04 (0.58–0.82)	0.69 ± 0.06 (0.59–0.79)	0.67 ± 0.05 (0.55–0.78)	0.72 ± 0.04 (0.59–0.82)
H7–H8	2.26 ± 0.09 (2.06–2.33)	2.17 ± 0.10 (1.94–2.33)	2.10 ± 0.08 (1.94–2.33)	2.26 ± 0.10 (1.94–2.33)	3.17 ± 0.11 (2.97–3.45)	3.19 ± 0.14 (2.86–3.50)	3.20 ± 0.12 (2.81–3.44)	3.18 ± 0.12 (2.99–3.46)	0.71 ± 0.05 (0.60–0.78)	0.68 ± 0.06 (0.56–0.82)	0.66 ± 0.05 (0.56–0.83)	0.71 ± 0.06 (0.56–0.78)
H8–H9	2.21 ± 0.08 (2.06–2.33)	2.12 ± 0.09 (1.94–2.33)	2.05 ± 0.08 (1.84–2.19)	2.22 ± 0.09 (2.06–2.33)	3.15 ± 0.11 (2.94–3.43)	3.17 ± 0.13 (2.87–3.43)	3.16 ± 0.12 (2.91–3.44)	3.15 ± 0.12 (2.87–3.33)	0.70 ± 0.05 (0.60–0.79)	0.67 ± 0.06 (0.57–0.81)	0.65 ± 0.04 (0.54–0.75)	0.71 ± 0.05 (0.62–0.81)
H9–H10	2.19 ± 0.08 (2.06–2.33)	2.09 ± 0.09 (1.84–2.33)	2.01 ± 0.08 (1.84–2.19)	2.18 ± 0.10 (1.84–2.33)	3.11 ± 0.11 (2.87–3.35)	3.14 ± 0.14 (2.80–3.66)	3.11 ± 0.12 (2.84–3.36)	3.11 ± 0.10 (2.90–3.34)	0.71 ± 0.05 (0.62–0.81)	0.67 ± 0.05 (0.54–0.83)	0.65 ± 0.04 (0.56–0.76)	0.70 ± 0.05 (0.56–0.79)

SL: Step length, SF: Step frequency, HP: high performance group, IP: intermediate performance group, LP: low performance group

Finally, no significant differences were observed between the groups in relation to the lead leg used during the race or in the curves in both sexes (*p* > 0.05).

## Discussion

The purpose of this study was to analyse the influence of SF and SL on final performance in 400 mH and to introduce a new variable—the SF/SL ratio—as a key performance indicator in this event. Typically, athletes increase both SF and SL to enhance speed, with SL generally being the most critical variable (Hanley et al., 2023; Otsuka and Isaka, 2019). The data in this study represent a significant sample of top performances from recent major championships, including the current men's world record and three women's world records, before being surpassed by Sydney McLaughlin at the 2024 Paris Olympics.

### Step Length

Elite athletes tend to take fewer steps during the race (Iskra, 2012). However, a lower number of steps is not the cause of better performance; rather, it is the superior physical condition of these athletes that leads to this reduction (Lindeman, 1995). The number of steps in each rhythmic unit, or what is known as the stride or step pattern, is not consistent throughout the race; it increases or decreases depending on the typology of pace changes made by the athlete. This race rhythm is individual, and there are multiple rhythmic structures among the world's top athletes (Casal et al., 2020; López et al., 2020). The first change in the pace usually occurs between the sixth and the eighth rhythmic unit, with lower-performing athletes making more frequent changes during the race (Iskra et al., 2021a, 2021b, 2022a). As shown in this study, the number of steps during the second half of the race significantly impacts final performance in both sexes, with significant differences between all performance groups, especially in the female sample, where a higher number of pace changes is usually observed. Additionally, step length was significantly correlated with the final performance across the entire sample. Therefore, the hypothesis that greater step length enhances performance in this discipline is accurate, though it must always consider individual characteristics (Iskra et al., 2021b). Furthermore, a reduction in the number of steps has been observed in recent years (Iskra et al., 2022b, 2024; Pietrzak and Iskra, 2016), corresponding to higher performance levels in the discipline, as also reflected in the data of this study.

Another factor to consider is the difference in step length between the first and the second 200-m interval, which is attributed to fatigue (Cicchella, 2022). In the 400-m flat race, this loss begins after the 200-m interval, becoming particularly pronounced after 300 m of the race (Hanon and Gajer, 2009). Although the 400 mH has a greater aerobic component, the decrease in step length is similar in the two events. However, no differences were found in the male sample in terms of the difference between the two halves of the race, while in the female sample, the HP group exhibited a slightly smaller decrease compared to the other two groups, as seen in previous studies (Guex, 2012). When analysing the intervals between hurdles separately, it can be observed that both men and women experienced a decrease in their average SL after the fifth hurdle, with a similar reduction between the finalists and the other athletes. However, the differences between these athletes were significant, once again highlighting the importance of this variable.

Moreover, when analysing step length considering the dominant leg, it should be noted that an even rhythmic structure will result in clearing alternate hurdles with each leg, while an odd rhythmic structure allows the same lead leg to be maintained for subsequent hurdles (Lindeman, 1995). Although attacking with the left leg offers biomechanical benefits and enables a shorter distance to be covered (Schiffer, 2012), no correlation was found with the final performance, nor were significant differences observed between groups. This may be due to the individual athlete’s preference for their dominant leg.

Therefore, analysing step length is crucial to understand performance in 400 mH, being a more critical variable for elite female athletes than for males. Additionally, analysing the second half of the race is essential (Pietrzak and Iskra, 2016).

### Step Frequency

Given the importance of optimising the number of steps per interval, it is logical to assume that step frequency is also crucial for performance. Both elite athletes and those at the national level experience a reduction in step frequency towards the end of the race, which is important to be minimised for maintaining the pace in both samples (Otsuka and Isaka, 2019). Additionally, step frequency appears to correlate well with final performance in athletes of various levels of performance (Guex, 2012). In the 400-m flat race, the reduction in SF becomes critical after 300 m, with a more pronounced decline in the final 50 m, which is greater than the loss in SL (Gajer et al., 2007; Hanon and Gajer, 2009; Mackala et al., 2024). In the first half of the race, athletes, especially women, tend to maintain the same number of steps between hurdles (Iskra et al., 2021b, 2022b), thus SF is the variable that determines whether speed is gained or lost. However, no significant differences were found in the first half of the race (Otsuka and Isaka, 2019), contradicting other research (Guex, 2012). This discrepancy could be due to a change in trends and a higher level of current athletes who, tending towards an effort distribution with a smaller decrease in speed at the end of the race—what is referred to as the “endurance” athlete typology (Iskra, 2012), perform a more similar first half of the race.

Lastly, in both male and female samples, there appears to be a prioritisation of SL over SF among higher-level athletes, with no significant differences between groups. When analysing the differences between the finalists and athletes who competed in the heats and semifinals, a trend towards lower SF in the final intervals was observed for the higher-level athletes. This is also related to greater SL and the effect of fatigue. Therefore, regarding the first objective of the study, it seems that step frequency alone may not be a reliable indicator of performance in elite 400 mH, requiring a more complex analysis in relation to other variables.

### SL/SF Ratio

Considering that the placement of the ten hurdles during the race affects both the SL and SF, athletes cannot run with an optimal relationship between these variables (Otsuka and Isaka, 2019). Therefore, a ratio between them has been proposed to allow for a more integrated interpretation (Guex, 2012). In the same study, a correlation was observed between the ratio throughout the race and during the second 200-m segment and the final performance. This value was found to be higher in high-performance athletes, although the analysis was conducted only on female athletes. Despite the lower step frequency values observed earlier, it is noticeable that the SL/SF ratio is higher in the HP group in both sexes. Additionally, significant differences were found between the HP group and the other groups in both sexes, both in the SL/SF ratio throughout the race and in the second 200-m segment.

Therefore, this variable is important for analysing performance in the 400 mH at an elite level, fulfilling the second objective of the study. This phenomenon can be attributed to the tendency of high-performing athletes to take fewer steps, which alters or decreases SF values in the latter half of the race. However, despite this reduction, the importance of SF should not be underestimated. Moreover, this study represents the first comparison between both sexes and, to our knowledge, it is the first study to include this variable in a male sample.

## Limitations

It should be noted that due to the observational nature of this study and the competitive context, it was not possible to obtain anthropometric data from athletes, which would influence SL or SF in the 400 mH. While the study's greatest strength lies in the use of in-competition cameras to analyse the world's top athletes, which yielded highly ecological results, the analyses do not possess the same sensitivity as those conducted in a laboratory setting. Given the competition situation, it was not possible to obtain the exact individual step length data for each athlete, necessitating the use of averages instead. Additionally, future research would benefit from including hurdle clearance data, which could provide insight into a broader range of kinematic variables. Finally, the characteristics of the sample, consisting of the world’s top athletes in competition, result in a smaller sample size than in other studies. Therefore, caution should be exercised when generalising these findings beyond the level of performance of these athletes.

## Practical Implications

SF is a crucial variable for performance in sprint events. However, the unique characteristics of the 400 mH require a distinct approach to its analysis. High-performance athletes appear to maintain speed through greater SL compared to lower-performing athletes, even if this results in a decrease in SF. Therefore, for training of the 400 mH, focusing on the interaction between both variables is crucial. To better understand the relationship between these variables, it seems beneficial to use a ratio that considers both SL and SF. This ratio enables a more in-depth analysis of the interaction between SL and SF in the 400 mH athlete’s performance. The finalists of both sexes maintained a higher SL/SF ratio throughout the race, with this difference being particularly significant towards the end of the race. Given their higher average SL and, consequently, fewer steps performed, it will be fundamental to adapt the step pattern to the one that allows each athlete to maintain the ratio between both variables with minimal loss during the final part of the race. Implementing this ratio in future studies is important, as analysing SF in isolation could lead to an underestimation of its significance.

## References

[ref1] Casal, N., López, J. L., Peña, I., Rodríguez, C., Oliván, J., Mateos, C., Balada, A., & Planas, A. (2020, October 28). Biomechanical analysis of the women’s 400 m hurdles at the IAAF World Athletics Championships Doha 2019: rhythmic structure and effort distribution. *25th European College of Sport Science Congress*.

[ref2] Cicchella, A. (2022). The Problem of Effort Distribution in Heavy Glycolytic Trials with Special Reference to the 400 m Dash in Track and Field. *Biology*, 11(2), 216. 10.3390/biology PMC8869504 35205083

[ref3] Duffield, R., Dawson, B., & Goodman, C. (2005). Energy system contribution to 400-metre and 800-metre track running. *Journal of Sports Sciences*, 23(3), 299–307. 10.1080/02640410410001730043 15966348

[ref4] Fritz, C. O., Morris, P. E., & Richler, J. J. (2012). Effect size estimates: Current use, calculations, and interpretation. *Journal of Experimental Psychology: General*, 141(1), 2–18. 10.1037/a0024338 21823805

[ref5] Gajer, B., Hanon, C., & Chantalle, M. (2007). Velocity and stride parametres in the 400 metres. *New Studies in Athletics*, 22(3), 39–46.

[ref6] Guex, K. (2012). Kinematic Analysis of the Women’s 400m Hurdles. *New Studies in Athletics*, 1(2), 41–51. http://www.degruyter.com/view/j/humo.2011.12.issue-4/v10038-011-0035-5/v10038-011-0035-5.xml

[ref7] Gupta, S., Goswami, A., & Mukhopadhyay, S. (1999). Heart rate and blood lactate in 400 M flat and 400 M hurdle running: A comparative study. *Indian Journal of Physiology and Pharmacology*, 43(3), 361–366. 10776485

[ref8] Hanley, B., Bissas, A., Merlino, S., & Burns, G. T. (2023). Changes in running biomechanics during the 2017 IAAF world championships men’s 1500 m final. *Scandinavian Journal of Medicine & Science in Sports*, 33(6), 931–942. 10.1111/sms.14331 36779698

[ref9] Hanon, C., & Gajer, B. (2009). Velocity and Stride Parameters of World-Class 400-Meter Athletes Compared With Less Experienced Runners. *Journal of Strength and Conditioning Research*, 23(2), 524–531. 10.1519/JSC.0b013e318194e071 19209080

[ref10] Hanon, C., Lepretre, P. M., Bishop, D., & Thomas, C. (2010). Oxygen uptake and blood metabolic responses to a 400-m run. *European Journal of Applied Physiology*, 109(2), 233–240. 10.1007/s00421-009-1339-4 20063105

[ref11] Hunter, J. P., Marshall, R. N., & Mcnair, P. J. (2004). Interaction of Step Length and Step Rate during Sprint Running. *Medicine & Science in Sports & Exercise*, 36(2), 261–271. 10.1249/01.MSS.0000113664.15777.53 14767249

[ref12] Iskra, J. (2012). Athlete Typology and Training Strategy in the 400m Hurdles. *New Studies in Athletics*, 27(1/2), 27–37.

[ref13] Iskra, J., & Coh, M. (2011). Biomechanical studies on running the 400 M hurdles. *Human Movement*, 12(4), 315–323. 10.2478/v10038-011-0035-5

[ref14] Iskra, J., Ćorluka, M., Vodicar, J., & Maćkała, K. (2021a). Extended analysis of types of stride patterns and pacing strategy in 400 m hurdle run. *Acta Kinesiologica*, 15(1), 15–23. 10.51371/issn.1840-2976.2021.15.1.2

[ref15] Iskra, J., Gupta, S., Przednowek, K., Best, R. V., & Stanula, A. (2024). The impact of physique on strategy and performance in the 400 m hurdles race among elite male athletes. *Baltic Journal of Health and Physical Activity*, 16(1), 4. 10.29359/BJHPA.16.1.04

[ref16] Iskra, J., Marcinów, R., & Walaszczyk, A. (2022a). Changes in the 400m hurdle run strategy after august 3,2021-revolution or just a progress. *Vedecká Konferencia Atletika* 2022, 20–28.

[ref17] Iskra, J., Matusiński, A., Otsuka, M., & Guex, K. J. (2021b). Pacing Strategy in Men’s 400 m Hurdles Accounting for Temporal and Spatial Characteristics of Elite Athletes. *Journal of Human Kinetics*, 79(1), 175–186. 10.2478/hukin-2021-0059 34400997 PMC8336559

[ref18] Iskra, J., Przednowek, K., Domaradzki, J., Coh, M., Gwiazdoń, P., & Mackala, K. (2022b). Temporal and Spatial Characteristics of Pacing Strategy in Elite Women’s 400 Meters Hurdles Athletes. *International Journal of Environmental Research and Public Health*, 19(6), 3432. 10.3390/ijerph19063432 35329119 PMC8956049

[ref19] Lindeman, R. (1995). 400 meter hurdle theory. *Track and Field Coaches Review*, 95, 33–36.

[ref20] López, J. L., & Casal, N. (2023). Análisis técnico y biomecánico de Sydney McLaughlin, plusmarquista mundial de 400 metros vallas, entre 2019 y 2022. *In: Generalitat de Catalunya editor. La Igualtat en Joc*. *Barcelona: INEFC Barcelona*.

[ref21] López, J. L., Casal, N., Peña, I., Rodríguez, C., Oliván, J., Mateos, C., Balada, A., & Planas, A. (2020, October 28). Biomechanical analysis of the men’s 400 m hurdles at the IAAF World Athletics Championships Doha 2019: rhythmic structure and effort distribution. *25th Congress European College of Sport Sciences*.

[ref22] Mackala, K., Omelko, R., Mroczek, D., Szczepan, S., & Mastalerz, A. (2024). Kinematics of Two Special Endurance Trials: A Methodological Contribution to 400-m Performance. *Journal of Human Kinetics*, 93, 181–191. 10.5114/jhk/185155 39132425 PMC11307176

[ref23] Mero, A., Komi, P. V., & Gregor, R. J. (1992). Biomechanics of Sprint Running. *Sports Medicine*, 13(6), 376–392. 10.2165/00007256-199213060-00002 1615256

[ref24] Mundt, M., Colyer, S., Wade, L., Needham, L., Evans, M., Millett, E., & Alderson, J. (2024). Automating Video-Based Two-Dimensional Motion Analysis in Sport? Implications for Gait Event Detection, Pose Estimation, and Performance Parameter Analysis. *Scandinavian Journal of Medicine & Science in Sports*, 34(7), e14693. 10.1111/sms.14693 38984681

[ref25] Nor Adnan, N. M., Ab Patar, M. N. A., Lee, H., Yamamoto, S. I., Jong-Young, L., & Mahmud, J. (2018). Biomechanical analysis using Kinovea for sports application. *IOP Conference Series: Materials Science and Engineering*, 342(1), 012097. 10.1088/1757-899X/342/1/012097

[ref26] Otsuka, M., & Isaka, T. (2019). Intra-athlete and inter-group comparisons: Running pace and step characteristics of elite athletes in the 400-m hurdles. *PLoS ONE*, 14(3), 1–17. 10.1371/journal.pone.0204185 PMC6438499 30921329

[ref27] Pietrzak, M., & Iskra, J. (2016). Changes in women’s 400 m hurdle run from 1978 to 2014. *Physical Activity Review*, 4, 132–138. 10.16926/par.2016.04.16

[ref28] Przednowek, K., Iskra, J., Maszczyk, A., & Nawrocka, M. (2016). Regression shrinkage and neural models in predicting the results of 400-metres hurdles races. *Biology of Sport*, 33(4), 415–421. 10.5604/20831862.1224463 28090147 PMC5143778

[ref29] Quercetani, R. (2000). *Athletics: A history of modern track & field athletics*. SEP Editrice.

[ref30] Salo, A. I. T., Bezodis, I. N., Batterham, A. M., & Kerwin, D. G. (2011). Elite Sprinting: Are athletes individually step frequency or step length reliant? *Medicine & Science in Sports & Exercise*, 43(6), 1055–1062. 10.1249/MSS.0b013e318201f6f8 20980924

[ref31] Schiffer, J. (2012). The 400 m Hurdles. *New Studies in Athletics*, 27(1–2), 9–24.

[ref32] Van der Meulen, L., Bonnaerens, S., Van Caekenberghe, I., De Clerq, D., Segers, V., & Fiers, P. (2024). Habitual Running Style Matters: Duty Factor, and Not Stride Frequency, Relates to Loading Magnitude. *Journal of Human Kinetics*, 94, 37–45. 10.5114/jhk/191528 39563764 PMC11571469

[ref33] Zouhal, H., Jabbour, G., Jacob, C., Duvigneau, D., Botcazou, T., Ben Abderrahaman, A., Prioux, J., & Moussa, E. (2010). Anaerobic and Aerobic Energy System Contribution to 400-M Flat and 400-M Hurdles Track Running. *Journal of Strength and Conditioning Research*, 24(9), 2309–2315. 10.1519/JSC.0b013e3181e31287 20703164

